# Could MR Angio Replace Digital Subtraction Angiography for Verification of Occlusion Rate of Cerebral Aneurysms?

**DOI:** 10.3390/jcm14228221

**Published:** 2025-11-20

**Authors:** Rasmus Moldt Holmager, Jonas Jensen, Troels Halfeld Nielsen, Sune Munthe

**Affiliations:** 1 Department of Neurosurgery, Odense University Hospital, 5000 Odense, Denmark; troels.nielsen@rsyd.dk (T.H.N.); sune.munthe@rsyd.dk (S.M.); 2 Department of Clinical Research and BRIDGE (Brain Research–Inter Disciplinary Guided Excellence), University of Southern, 5000 Odense, Denmark; 3 Department of Radiology, Aarhus University Hospital, 8200 Aarhus N, Denmark; jonas.jensen@rm.dk

**Keywords:** intracranial aneurysm, magnetic resonance angiography, digital subtraction angiography, endovascular treatment, follow-up imaging

## Abstract

**Background/Objectives:** Cerebral aneurysms affect 3–5% of the population and carry a risk of rupture. Post-treatment imaging is essential to detect residual or recurrent filling. Digital subtraction angiography (DSA) is the gold standard, but it is invasive and can have complications. Magnetic resonance angiography (MRA) is a non-invasive alternative, but its reliability in follow-up remains debated. This study evaluated the diagnostic performance of MRA compared to DSA in detecting residual flow after endovascular treatment of intracranial aneurysms. Primary outcomes were MRA’s sensitivity, specificity, positive predictive value (PPV), and negative predictive value (NPV) and its potential to reduce or replace DSA in follow-up. **Methods:** A retrospective single-center study included patients ≥ 18 years treated endovascularly for ruptured or unruptured intracranial aneurysms during 2018–2021. Each follow-up case with both DSA and MRA was independently assessed by neuroradiologists, using DSA as the reference standard. Diagnostic performance metrics were calculated. **Results:** Of 400 screened cases, 198 were included. MRA and DSA findings agreed in 78.3% of cases. MRA showed 69.7% sensitivity and 80.0% specificity. PPV was 41.1%, and NPV was 93.0%. There were 33 false positives and 10 false negatives. Only one false negative led to retreatment, and none of the false positives required intervention. **Conclusions:** MRA shows high reliability in ruling out residual aneurysm flow due to its strong NPV. Despite limitations in confirming recurrence, MRA might be a useful first-line follow-up tool, with DSA reserved for inconclusive, unclear, or artifact cases.

## 1. Introduction

The prevalence of cerebral aneurysms in the general population is estimated at 3–5% [1], and the annual cumulative risk of aneurysm rupture is around 1.4%, although this varies depending on factors such as aneurysm size, location, growth and age [2,3]. Rupture resulting in subarachnoid hemorrhage (SAH) is associated with a high case fatality, with approximately one-third of patients dying within the first month, and poor functional outcome reported in about 27% of survivors at one month [4]. Endovascular treatment is commonly used for both ruptured and unruptured intracranial aneurysms but is not without risk, while alternative treatments also have their advantages and disadvantages [5,6,7].

Due to the risk of rebleeding or rupture of the treated aneurysm, patients need medical follow-up within the first 18 months after occlusion [8,9]. Later retreatment is more likely after endovascular treatment, while retreatment after surgical clipping mainly occurs earlier than for patients treated with endovascular coiling [9].

Digital subtraction angiography (DSA) and magnetic resonance angiography (MRA) are used to examine if the treated aneurysm has been sufficiently closed [10,11]. While DSA is the gold standard for evaluating aneurysm occlusion after endovascular treatment, its invasive nature limits its routine use and increases the risk of complications such as embolic stroke, groin hematoma, and contrast-induced nephropathy, and it can only be performed at selected hospitals and radiology departments [8,12]. A study in 2022 showed an overall complication rate of 5%; permanent neurological complications are rare but occur in 1% of DSA cases and are more common in patients over 55 years old [12,13]. In contrast, MRA is non-invasive, widely available, and avoids ionizing radiation and iodinated contrast. However, the diagnostic accuracy of MRA can vary depending on scanner field strength, pulse sequences, and the use of devices such as stent-assisted coils and flow diverters, which can cause susceptibility and signal-loss artifacts that may obscure residual aneurysm filling [14,15].

At our department of neurosurgery, it is standard practice to perform both DSA and MRA, with DSA serving as the reference. Lane et al. [16] provided evidence supporting the use of MRA instead of DSA for detecting recurrent aneurysms after endovascular treatment. But no such evidence is yet available or validated regarding the follow-up of patients in Denmark, nor has it been investigated whether the increased use of stent-assisted coiling and flow diverters influences the choice of MRA over DSA. Furthermore, Schaafsma et al. [8] demonstrated that MRA offers economic advantages while achieving health outcomes comparable to those of DSA.

Therefore, clarification of the diagnostic reliability of MRA in routine clinical follow-up, including patients treated with stent-assisted coiling and flow diversion, is of importance.

This retrospective study aimed to determine the sensitivity, specificity, and positive and negative predictive value of MRA compared to DSA for determining the occlusion rate of endovascularly treated intracranial aneurysms. If MRA is found to be superior to DSA, it may allow for easier post-treatment follow-up for both patients and staff, and fewer health risks for patients.

## 2. Materials and Methods

### 2.1. Study Design and Population

This retrospective single-center study was conducted at the Department of Neurosurgery, Odense University Hospital, Denmark. The study group comprised all patients aged 18 years or older who had undergone endovascular treatment for ruptured or unruptured intracranial aneurysms between 1 January 2018 and 31 December 2021. Exclusion criteria comprised patients who did not undergo both DSA and MRA at the follow-up after treatment, which was presumed to reflect factors such as death, poor clinical condition, imaging artifacts, or the use of Vaso-CT instead of DSA.

The DSA images (all biplane acquisitions) were assessed by different neuroradiologists working independently. The original DSA reports issued shortly after each examination at the follow-up time were used in the analysis. To avoid the same neuroradiologist evaluating both the DSA and the corresponding MRA, all MRA images (all MR Time-of-Flight [TOF] sequences) were subsequently re-evaluated by an external, blinded neuroradiologist from another institution, who was unaware of the DSA findings, and thereby minimizing potential bias.

The MRA scans were performed predominantly on a 1.5T system. For all examinations, the imaging parameters were TR/TE = 25/6.9 ms, voxel size = 0.5 × 0.87 mm, and slice thickness = 0.5 mm. The image descriptions of the DSA and the MRA were recorded in an external document, noting whether there was continuous flow or total occlusion at the treated aneurysm. Each imaging evaluation of an endovascularly treated aneurysm was documented as a separate case, so patients with multiple treated aneurysms could be represented by multiple cases. Additionally, an aneurysm that underwent retreatment and subsequent follow-up imaging during the inclusion period could have multiple follow-up assessments, leading to multiple cases for the same aneurysm.

The Raymond–Roy Occlusion Classification (RROC), also known as the Montreal scale [17], and its three grading levels were not used by the neuroradiologists to assess the treated aneurysm. However, for analytical purposes, the aneurysms were classified according to two groups: class 1 was considered complete occlusion, while classes 2, 3a, and 3b were grouped together as indicating any degree of residual flow. The DSA was used as the reference modality. Any documented reasons for missing scans or rejection of treatment were noted.

### 2.2. Data Collection

All data, including the radiology scans, were collected from the electronic patient journals.

### 2.3. Data Storage and GDPR

Data were stored in the online database SharePoint, ensuring that handling of personal information complied with national laws and the guidelines of the Danish Health Care Act. Data were collected and stored exclusively by study personnel with data locking.

### 2.4. Data Processing and Analysis

Continuous variables are presented as means ± standard deviation (SD) if normally distributed. Non-normally distributed variables are presented as medians with interquartile ranges (IQR). Categorical data are presented as absolute numbers and percentages. The normality of continuous variables was assessed using the Shapiro–Wilk test.

The data were registered and processed in Excel and then analyzed in both Excel and MedCalc^®^ using the ‘Diagnostic test evaluation calculator’. Observations of true positives, true negatives, false positives, and false negatives were used to determine sensitivity, specificity, positive predictive values, and negative predictive values with 95% confidence intervals (95% CI).

## 3. Results

**Figure 1:**
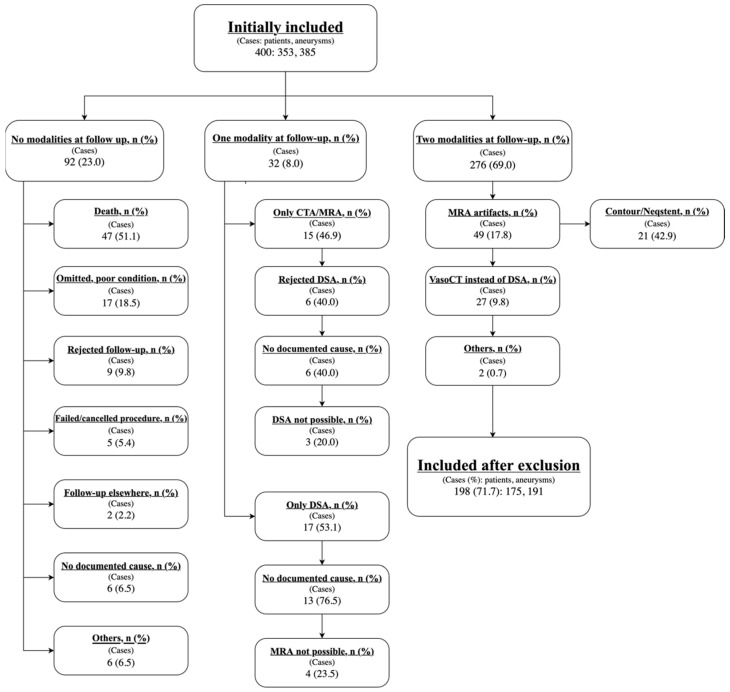
Flowchart of the cases included and reasons for exclusion. MRA: Magnetic resonance angiography, DSA: Digital subtraction angiography, VasoCT: Cone-beam computed tomography in interventional radiology.

An initial total of 400 cases (comprising 353 patients and 385 aneurysms) had undergone endovascular treatment from 1 January 2018 to 31 December 2021. Of these, 276 had both DSA and MRA imaging at follow-up. 202 cases were excluded mainly due to death, poor condition and MRA scanning artifacts, for example, from Contour and Neqstent. For further information regarding exclusions, see Figure 1.

A total of 198 cases (175 patients and 191 treated endovascular aneurysms) were included in data analysis. Table 1 shows their demographic and clinical characteristics. In 195 of the 198 cases, the MRA and DSA examinations were performed on the same day. In the remaining three cases, the interval between the two modalities was 5, 14, and 30 days, respectively.

For 155 (78.3%) of the 198 cases, we found agreement between DSA and MRA, where 132 (85.2%) cases showed no signs of recurrence on either DSA or MRA, and 23 (14.8%) cases showed continuous flow/filling of the aneurysm on both DSA and MRA. For 43 (21.7%) of the 198 cases, we found a discrepancy between DSA and MRA, where 33 cases showed a small aneurysm recurrence on the MRA that was not seen on the corresponding DSA (none of these cases led to further treatment or additional follow-up), and 10 cases showed continuous aneurysmal flow on the DSA but not on the MRA. One of these cases resulted in retreatment, which led to complete occlusion; in another case, follow-up DSA performed two years later showed unchanged findings, and the patient was subsequently discharged; the remaining eight cases required neither further treatment nor additional follow-up.

Using the DSA as the gold standard for detecting recurrent aneurysmal flow, the overall sensitivity of MRA was 69.7% (95% CI: 51.3–84.4%) and the specificity was 80.0% (95% CI: 73.1–85.8%). The positive predictive value was 41.1% (95% CI: 32.3–50.5%), and the negative predictive value was 93.0% (95% CI: 88.7–95.7%). For further results in the subgroups, see Table 2.

## 4. Discussion

The study findings demonstrate that MRA has a high negative predictive value, suggesting its reliability in ruling out residual aneurysm flow. The overall sensitivity and specificity for MRA compared to DSA were 69.7% and 80.0%, respectively, while the negative predictive value reached 93.0%. This indicates that MRA may be useful as an initial screening tool, particularly for patients with a low risk of recurrence. A negative MRA could potentially eliminate the need for further DSA in these patients, thus optimizing follow-up protocols and reducing exposure to the risks associated with the invasive DSA.

However, MRA demonstrated a relatively low positive predictive value of 41.1%, indicating that a positive result does not reliably confirm true aneurysm recurrence. This is particularly notable in the ruptured subgroup, where the positive predictive value was as low as 48.3%. Special attention is warranted for previously ruptured aneurysms as they are associated with an increased risk of regrowth and subsequent re-rupture. Therefore, it is essential to accurately assess whether residual flow persists within the aneurysm, highlighting the necessity for additional imaging to resolve diagnostic uncertainty.

A key observation in this study was the discrepancy between MRA and DSA findings in 43 cases, comprising 33 false positives and 10 false negatives. The predominance of false-positive findings suggests that MRA may overestimate residual aneurysmal flow. Conversely, the 10 false-negative cases underscore the potential for MRA to underestimate residual aneurysm filling.

The overestimation likely reflects inherent technical limitations of MRA, such as susceptibility artifacts, flow-related signal alterations, and thrombus-associated hyperintensities, which can mimic residual flow. Notably, none of these cases led to unnecessary retreatment, suggesting limited clinical impact [14,18,19]. Underestimation tends to occur in small or slow-flow remnants, where MRA sensitivity is reduced due to limited resolution and insensitivity to low or complex flow patterns, particularly in the presence of metallic implants [14,18]. Nonetheless, with only one of these cases requiring further intervention, the clinical impact of false-negative findings appears to be limited. Taken together, these factors appear to represent the primary risk factors for false-positive and false-negative findings, respectively. These results align with the meta-analysis by van Amerongen et al. [20] from 2014 and the more recent one from Ahmed et al. [21] from 2019, which demonstrated high sensitivity and specificity for MRA in detecting residual or recurrent aneurysms. Specifically, van Amerongen et al. [20] reported a sensitivity of 86% (95% CI: 82–89%) and a specificity of 84% (95% CI: 81–88%) for TOF MRA, while Contrast-Enhanced (CE) MRA demonstrated a sensitivity of 86% (95% CI: 82–89%) and a specificity of 89% (95% CI: 85–92%). Similarly, Ahmed et al. [21] reported slightly higher values, with TOF MRA at 88% and 94%, and CE MRA at 88% and 96%. When compared with these two meta-analyses [20,21], the present study demonstrated slightly lower sensitivity and specificity, possibly reflecting differences in study design, imaging protocols, or patient characteristics.

The practical implication of these findings suggests a stratified approach to aneurysm follow-up. MRA can be employed as the primary screening modality, with DSA reserved for cases where MRA findings are inconclusive or suggest aneurysm recurrence. If uncertainty remains regarding MRA results, especially in cases of continued residual flow, referral to DSA is recommended. Special attention should be given to certain patient groups, such as those with Contour stents, Neqstents, or other artifact-generating materials that may interfere with MRA interpretation. Artifacts related to stent-assisted coiling and flow diverters were a major cause of non-diagnostic MRA results, observed in 49 cases, including 21 with Contour or Neqstent devices. These artifacts arise from magnetic interference caused by the implanted materials and can mask residual flow, thereby reducing the diagnostic reliability of MRA in assessing aneurysm occlusion. Although the present study did not quantify the extent of these artifacts, their impact on image interpretability highlights the need for careful patient selection and supports the use of DSA in cases with suspected device-related artifacts. Furthermore, patients with known incomplete aneurysm occlusion from the initial treatment, where residual flow is expected, should also be considered for more comprehensive follow-up with DSA.

From a healthcare perspective, the use of MRA as a first-line modality for follow-up of treated intracranial aneurysms may also have economic advantages. Previous studies, such as Schafmann et al. [8], have shown that replacing DSA with MRA in selected cases can considerably reduce overall costs. Although the present study did not include a detailed cost analysis, it is reasonable to assume that a similar benefit could be achieved in a Danish context. In addition to potential financial savings, the use of MRA may also reduce patient transport time and logistical burden, as MRA can often be performed at local hospitals, whereas DSA is typically limited to larger hospitals.

The current study has several limitations. The exclusion of a significant number of cases due to imaging artifacts, primarily caused by Contour stents and Neqstents, underscores a technical challenge in using MRA for follow-up. Additionally, the lack of standardized occlusion grading, such as the Raymond–Roy Occlusion Classification, limits the specificity of the findings. Future studies should incorporate a standardized grading system and explore the economic and logistical benefits of replacing DSA with MRA in specific patient populations, and should also aim to include at least two independent, blinded neuroradiologists for the interpretation of each imaging modality. Furthermore, ongoing developments in MR angiography sequences and post-processing techniques, such as higher field strength imaging, optimized time-of-flight protocols, and advanced artifact reduction methods, may further improve the diagnostic reliability of MRA for verification of aneurysm occlusion.

## 5. Conclusions

The findings of this study demonstrate that MRA has a high reliability in excluding residual aneurysm flow due to its high negative predictive value. Although the lower positive predictive value limits its ability to confirm recurrence, the clinical impact of false positives appears minimal. These findings support the use of MRA as a first-line screening tool, with DSA reserved for cases with inconclusive MRA results or continuous residual flow. The findings also suggest that patients treated with Contour stents and Neqstents should be offered DSA as the first choice due to an above-normal frequency of artifacts on MRA.

This study indicates that follow-up imaging may be optimized by differentiating the modality according to the implanted device. However, the findings should be interpreted with caution due to artifact-related exclusions, and future research should incorporate standardized grading and evaluate newer MRA techniques to clarify its role as a primary follow-up modality. Further evaluation of workflow, cost, and patient impact is warranted to clarify the role of MRA as a primary follow-up modality.

## Figures and Tables

**Table 1:** Demographic and clinical characteristics of 198 cases of intracranial aneurysms that underwent endovascular treatment.

	Included in Analysis (*n* = 198)
Males cases, *n* (%)	61 (30.8)
Female cases, *n* (%)	137 (69.2)
Age at treatment, mean (SD)	56.8 (11.5)
Male cases	55.7 (12.5)
Female cases	57.3 (11.0)
Aneurysm rupture status, *n* (%)	
Ruptured aneurysms	88 (44.4)
Unruptured aneurysms	85 (42.9)
Planned retreatment, unruptured	25 (12.6)
Aneurysm size, mm^3^, median (IQR)	99.5 [36.0–367.5]
Missing or insufficient data	44
Months from treatment to follow-up, median (IQR)	11.0 [8.8–17.3]
Ruptured aneurysms	9.2 [8.5–10.3]
Unruptured aneurysms	14.5 [9.3–18.1]
Planned retreatment, unruptured	17.4 [16.3–18.7]
Location of treated and retreated aneurysm, *n* (%)	
MCA	16 (8.1)
Basilar bifurcation	14 (7.1)
ICA	41 (20.7)
Posterior communicating	29 (14.6)
Anterior communicating	64 (32.3)
Posterior circulation (others)	23 (11.6)
Anterior circulation (others)	11 (5.6)

SD: standard deviation, IQR: Interquartile range, MCA: Middle cerebral artery, ICA: Internal carotid artery.

**Table 2:** Diagnostic test evaluation for ruptured and unruptured aneurysms, retreatment, and overall.

	Sensitivity % (95% CI)	Specificity % (95% CI)	PPV % (95% CI)	NPV % (95% CI)
Ruptured	77.8 (52.4–93.6)	78.6 (67.1–87.5)	48.3 (35.9–60.9)	93.2 (85.2–97.1)
Unruptured	58.3 (27.7–84.8)	83.6 (73.1–91.2)	36.8 (22.4–54.1)	92.4 (86.1–96.0)
Retreatment	66.7 (9.4–99.2)	72.7 (49.8–89.3)	25.0 (10.4–48.8)	94.1 (76.0–98.8)
Overall	69.7 (51.3–84.4)	80.0 (73.1–85.8)	41.1 (32.3–50.5)	93.0 (88.7–95.7)

95% CI: 95% confidence interval. PPV: positive predictive value. NPV: negative predictive value.

## Data Availability

All data are available upon request to the corresponding author.
